# Successful Endoscopic Removal and Closure of a Large Esophageal Perforation Following Accidental Ingestion of a Dental Prosthesis

**DOI:** 10.1002/deo2.70270

**Published:** 2026-01-20

**Authors:** Takashi Akutagawa, Daisuke Yamaguchi, Moeko Shirouzu, Yutaro Fujimura, Motoaki Yuhi, Shohei Matsufuji, Yukie Yoda, Motohiro Esaki

**Affiliations:** ^1^ Department of Endoscopic Diagnostics and Therapeutics Saga University Hospital Saga Japan; ^2^ Division of Gastroenterology Department of Internal Medicine Faculty of Medicine Saga University Saga Japan; ^3^ Department of Surgery Faculty of Medicine Saga University Saga Japan

**Keywords:** endoscopic closure, endoscopic removal, esophageal perforation, foreign body ingestion, OTSC

## Abstract

Endoscopic removal of accidentally ingested dental prostheses can be challenging as their irregular shapes can occasionally cause severe complications, such as gastrointestinal perforation. Here, we present the case of an older woman who was referred to our hospital following accidental ingestion of a bridge‐type denture. Computed tomography revealed that the denture was lodged in the thoracic esophagus, with concurrent mediastinal emphysema. Endoscopic examination confirmed that the denture had penetrated the esophageal wall. Under general anesthesia, the denture was endoscopically removed using dual endoscopes, and a large esophageal perforation was closed with an over‐the‐scope clip (OTSC) and subsequently reinforced with Mantis clips. Although follow‐up endoscopy 1 month later demonstrated remaining OTSC at the site of the esophageal perforation, 3‐month follow‐up endoscopy confirmed complete closure of the perforation. Overall, this case indicates the usefulness of the dual‐endoscope approach for foreign‐object removal and the OTSC system for closure of esophageal perforations, thus providing the chance of avoiding invasive treatment such as esophagectomy.

## 1 Introduction

Foreign body ingestion is not a rare clinical problem; however, most cases involve small objects such as coins or buttons [1]. In contrast, dental prostheses present unique challenges owing to their size and irregular shape. Among these, bridge‐type dentures pose significant challenges in medical management and may require specialized interventions for safe extraction [2]. Denture ingestion can lead to severe complications, including gastrointestinal perforation [3]. An over‐the‐scope clip (OTSC) device, equipped with a bear‐trap‐like clip mounted on the applicator cap of an endoscope, has previously proven to be an effective tool for closing large perforations in the gastrointestinal tract [4, 5]. Herein, we report a case of esophageal perforation caused by accidental ingestion of a bridge‐type denture, successfully managed by an endoscopic procedure.

## 2 Case Report

**FIGURE 1:**
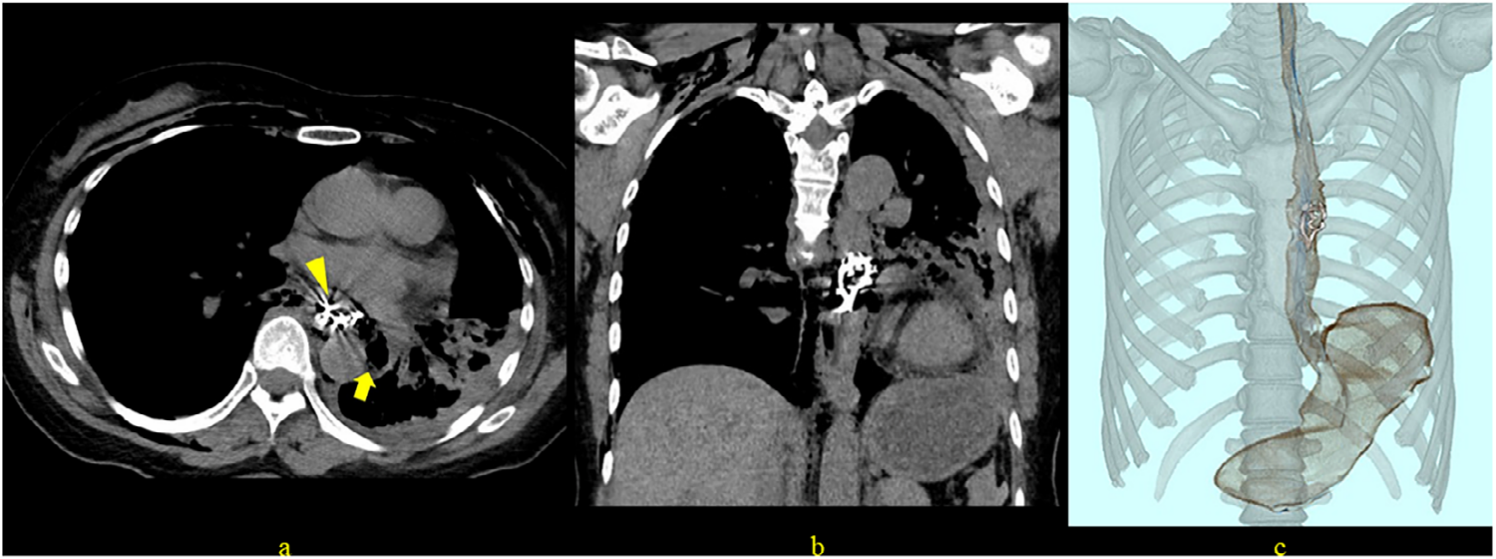
Computed tomography (CT) images at admission. (a) Axial view showing a high‐density structure located in the esophagus (arrowhead) with mediastinal emphysema (arrow). (b) Coronal view revealing the longitudinal extent of the impacted denture. (c) 3D reconstruction image clarifying the shape of the bridge‐type denture and its positional relationship with the surrounding structures.

The patient was a woman in her 60s without any significant medical history, including dementia, who visited another hospital because of suspicion of swallowing her bridge‐type partial denture during a meal. An emergent esophagogastroduodenoscopy (EGD) revealed a denture lodged in the thoracic esophagus. However, as the removal attempt was unsuccessful, the patient was referred to our hospital for further management. On arrival at our hospital, she complained of chest pain. Physical examination revealed no fever. Laboratory tests showed a white blood cell (WBC) count of 9100/µL, while the C‐reactive protein (CRP) level was 0.16 mg/dL. Computed tomography (CT) of the chest subsequently revealed a high‐density structure consistent with the missing denture lodged in the thoracic esophagus (Figure 1). The examination also showed mediastinal emphysema, suggesting esophageal perforation.

**FIGURE 2:**
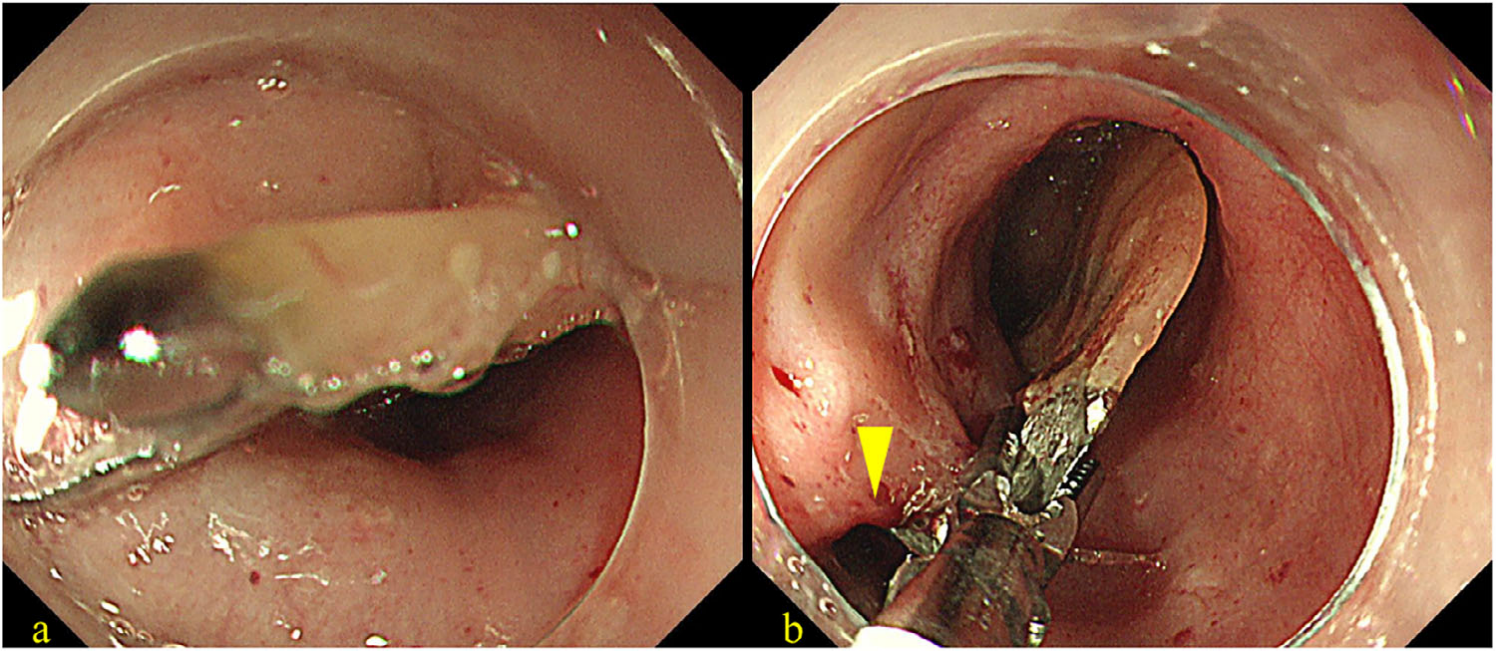
Preoperative endoscopic images showing (a) bridge‐type denture piercing the esophageal wall and (b) the perforation site in the esophageal wall (arrowhead).

**FIGURE 3:**
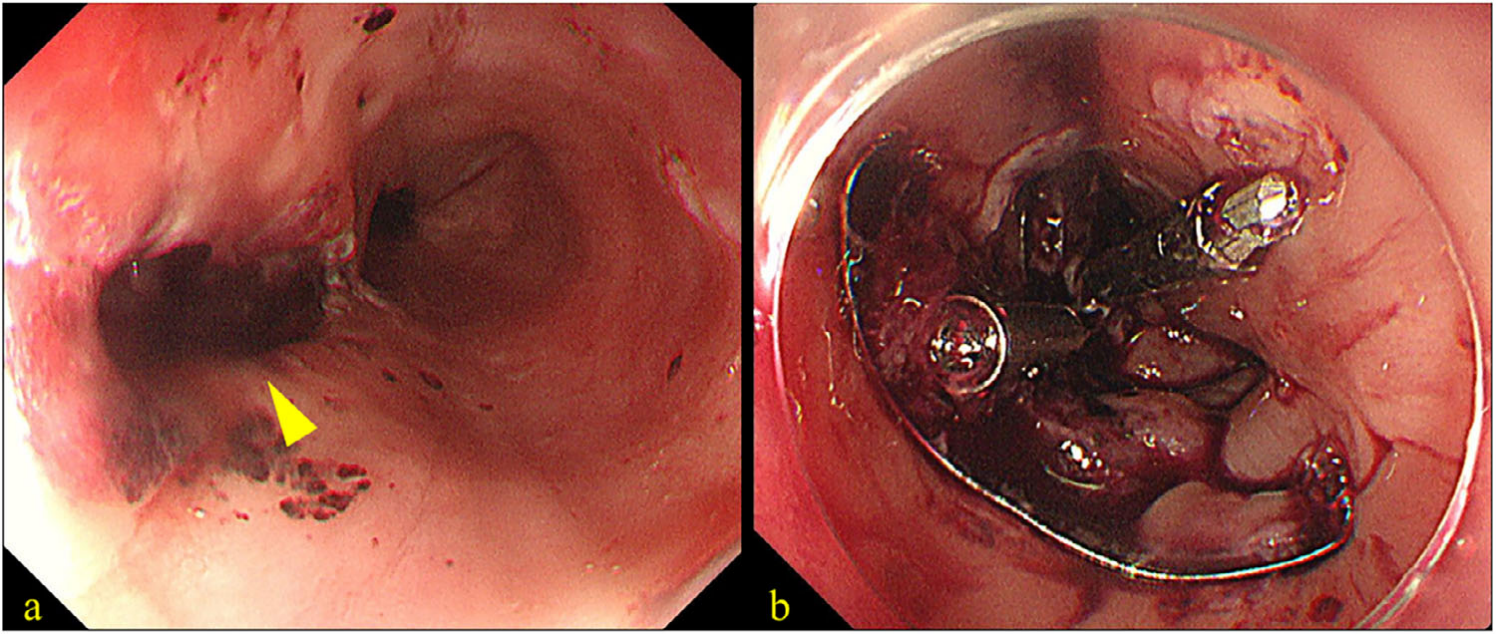
Images taken during endoscopic closure. (a) Perforation site observed after removal of the denture (arrowhead). (b) Over‐the‐scope clip (OTSC) and Mantis clips used to reinforce the perforation closure.

**FIGURE 4:**
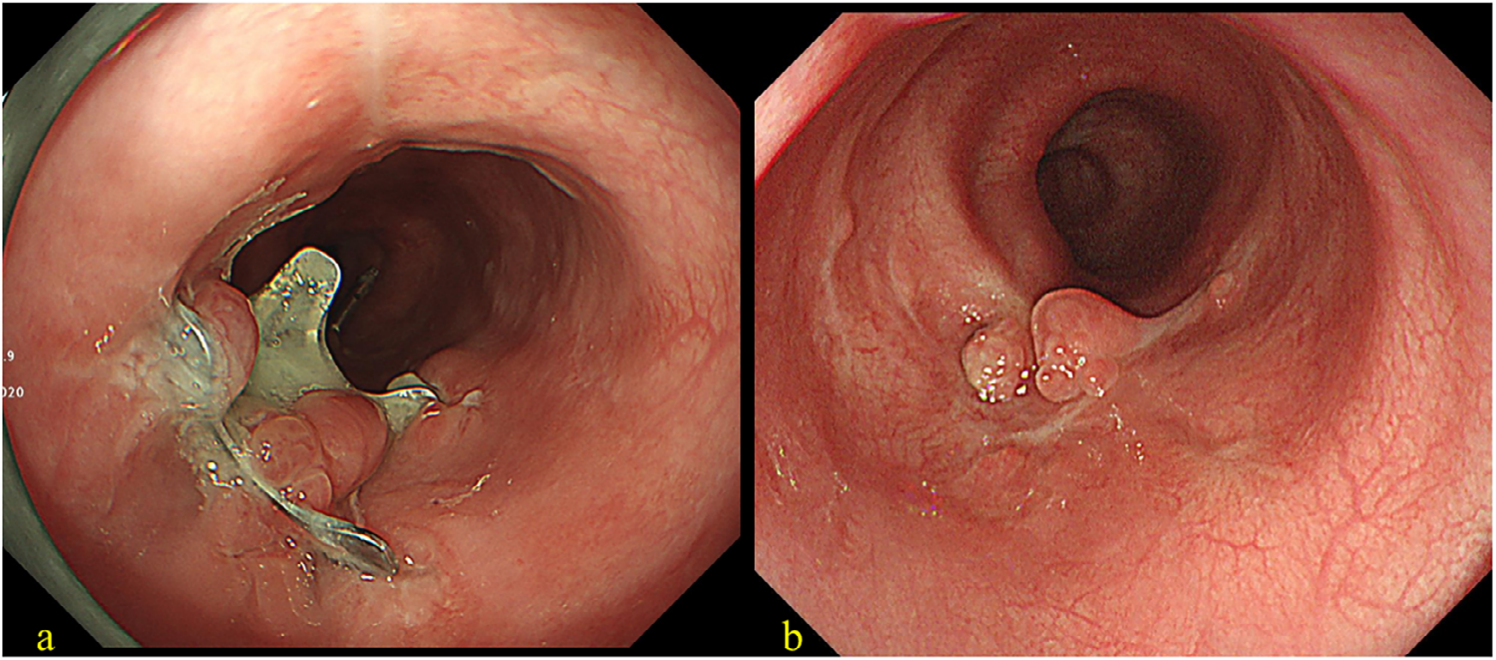
Postoperative endoscopic images. (a) One‐month follow‐up image showing the remaining over‐the‐scope clip (OTSC). (b) Three‐month follow‐up image showing complete closure of the perforation.

Urgent endoscopic examination revealed a bridge‐type denture penetrating the thoracic esophageal wall (Figure 2a), resulting in a visible perforation (Figure 2b). Although surgical removal was considered, endoscopic removal of the denture and closure of the esophageal perforation was ultimately attempted under general anesthesia. A dual‐endoscope approach was employed using a standard gastrointestinal scope (GIF‐H290T; Olympus Corp., Tokyo, Japan) and an ultrathin gastrointestinal scope (EG‐L580NW7; Fujifilm Corp., Tokyo, Japan). As shown in Figure S1, each end of the denture was grasped using endoscopic forceps, released from the penetration site, and carefully maneuvered into the stomach. After inserting the overtube, the denture was again grasped using two endoscopes and carefully withdrawn from the mouth (Video S1). We subsequently performed endoscopic closure of the esophageal perforation (Figure 3a). Given the large perforation, we first targeted the oral side of the defect using the OTSC system (Ovesco Endoscopy AG, Tübingen, Germany). However, the anal side of the perforation remained patent after OTSC deployment. Therefore, we placed two additional Mantis clips (Boston Scientific Japan, Tokyo, Japan) on the anal side to achieve complete closure of the remaining defect (Figure 3b and Video S1). The time required for the closure was 25 min. The patient was subsequently managed under nothing per os. Antibiotic therapy with ampicillin/sulbactam was administered for 3 weeks. The WBC count peaked at 13,000/µL, and the CRP level increased to 26 mg/dL on postoperative day 2. Although the patient exhibited fever for the first 3 days after the procedure, she became afebrile from day 4. Enteral nutrition via a nasogastric tube was initiated on postoperative day 9. Follow‐up endoscopy performed 1 month later revealed that the OTSC remained securely in place at the perforation site (Figure 4a), and a contrast study confirmed only minimal leakage. Therefore, oral intake was initiated. The patient's clinical course was uneventful, and she was discharged 54 days after admission. The detailed clinical course is presented in Figure S2. A subsequent endoscopy conducted 3 months after the procedure confirmed complete closure of the esophageal wall (Figure 4b).

## 3 Discussion

The ingestion of foreign bodies is a somewhat common clinical occurrence, with most objects being small and passing through the gastrointestinal tract without any complications [1]. However, the ingestion of dental prostheses, particularly bridge‐type dentures, poses a significant challenge owing to their problematic size and shape. These characteristics increase the risk of severe complications, including impaction and gastrointestinal perforation, which can be life‐threatening [6].

Esophageal perforation represents a serious medical emergency. Traditionally, large perforations require surgical management, ranging from primary repair to more radical procedures such as esophagectomy, both of which are associated with significant morbidity and mortality [7]. However, advances in endoscopic techniques have allowed the use of less invasive alternatives for managing such cases. Among these, the OTSC system represents a notable advancement. This device features a large, bear‐trap‐like clip, enabling the secure grasping and closure of sizable gastrointestinal wall defects. The efficacy of the OTSC system in treating large perforations, fistulas, and bleeding has been well‐documented in several studies and reviews [4, 8]. Specifically, a systematic review reported an overall clinical success rate of 57%–100% (pooled estimate: 80%) for gastrointestinal perforations, including esophageal cases [8].

In the present case, successful endoscopic management of an esophageal perforation caused by an impacted bridge‐type denture was achieved through the following two‐step procedure: endoscopic removal using the dual‐endoscope approach, and closure with the OTSC and additional Mantis clips. Bridge‐type dentures can be difficult to retrieve, as they often have sharp edges [9]. However, using dual endoscopes, both edges can be grasped securely, enabling safe removal. Thus, the dual‐endoscope approach is considered a valuable technique. Regarding closure, the esophagus is a narrow tubular organ located in the mediastinum, and its wall has limited mobility. Thus, it is sometimes difficult to close large wall defects using the OTSC system alone [5, 10]. In cases such as ours, the combination of OTSC and Mantis clips may be helpful.

Besides the OTSC system, several other endoscopic techniques are available for managing esophageal perforations. The Reopenable Clip‐Over‐the‐Line Method (ROLM) allows the closure of large defects by approximating the wound edges using clips and a line, whereas endoscopic hand suturing enables direct and secure closure. However, both techniques require advanced technical skills and precise maneuvering, often resulting in longer procedural times. Self‐expandable metal stents are also effective for sealing perforations but are associated with the risk of migration and stricture formation. By contrast, the OTSC system allows for simple and secure full‐thickness closure of larger defects in a single session, making it a suitable choice for this case.

This endoscopic approach has a few limitations. The primary indications for endoscopic closure include clinically stable patients with early diagnosis and contained leaks without widespread contamination. Contraindications include hemodynamic instability, septic shock, and extensive mediastinal or pleural fluid collections requiring surgical drainage. Furthermore, extremely large perforations exceeding the size of the OTSC cap, those with necrotic edges, or cases with concomitant severe infections such as abscesses may not be amenable to this technique and would require surgical intervention to achieve closure [11].

In the present case, this minimally invasive endoscopic approach allowed the patient to avoid major surgical procedures. Her uneventful postoperative outcomes, confirmed by serial follow‐up endoscopies showing complete healing, underscore the clinical value and potential of this minimally invasive approach. Based on our experience, we propose that this endoscopic management strategy, comprising the dual‐endoscope approach with the OTSC system, is a feasible and effective treatment option for the removal of dental prosthesis, particularly for bridge‐type dentures.

## Author Contributions

**Takashi Akutagawa**, **Daisuke Yamaguchi**, **Moeko Shirouzu**, **Yutaro Fujimura**, **Motoaki Yuhi**, **Shohei Matsufuji**, and **Yukie Yoda** were responsible for the patient's care. **Takashi Akutagawa** wrote the original draft. **Motohiro Esaki** supervised the study and critically revised the manuscript.

## Conflicts of Interest

Motohiro Esaki is an Associate Editor of *DEN Open*. The other authors declare no conflicts of interest.

## Funding

This study did not receive any funding.

## Ethics Statement

N/A

## Supporting information




**Figure S1**: Schematic illustration of the dual‐endoscope approach. The image depicts the bridge‐type denture being grasped using two endoscopes.

**Figure S2**: Clinical course summarizing the timeline of symptoms, treatment procedures, and laboratory data (C‐reactive protein [CRP] level and white blood cell [WBC] count).

**Video S1**: The bridge‐type denture was endoscopically extracted, and the perforation was effectively closed using OTSC and Mantis clips.
