# Supplementary figures and images for: Successful Endoscopic Removal and Closure of a Large Esophageal Perforation Following Accidental Ingestion of a Dental Prosthesis
Source: DEN Open. 2026 Jan 20;6(1):e70270. doi: 10.1002/deo2.70270 (PMC12819163; doi:10.1002/deo2.70270)

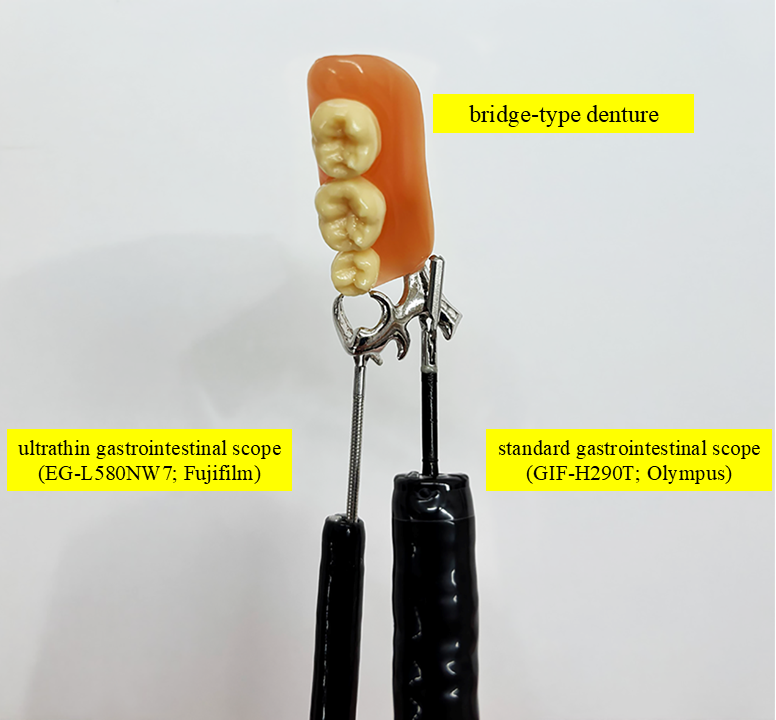

Supplement: Supplementary file 1 — Figure S1: Schematic illustration of the dual‐endoscope approach. The image depicts the bridge‐type denture being grasped using two endoscopes. [file DEO2-6-e70270-s003.tif]

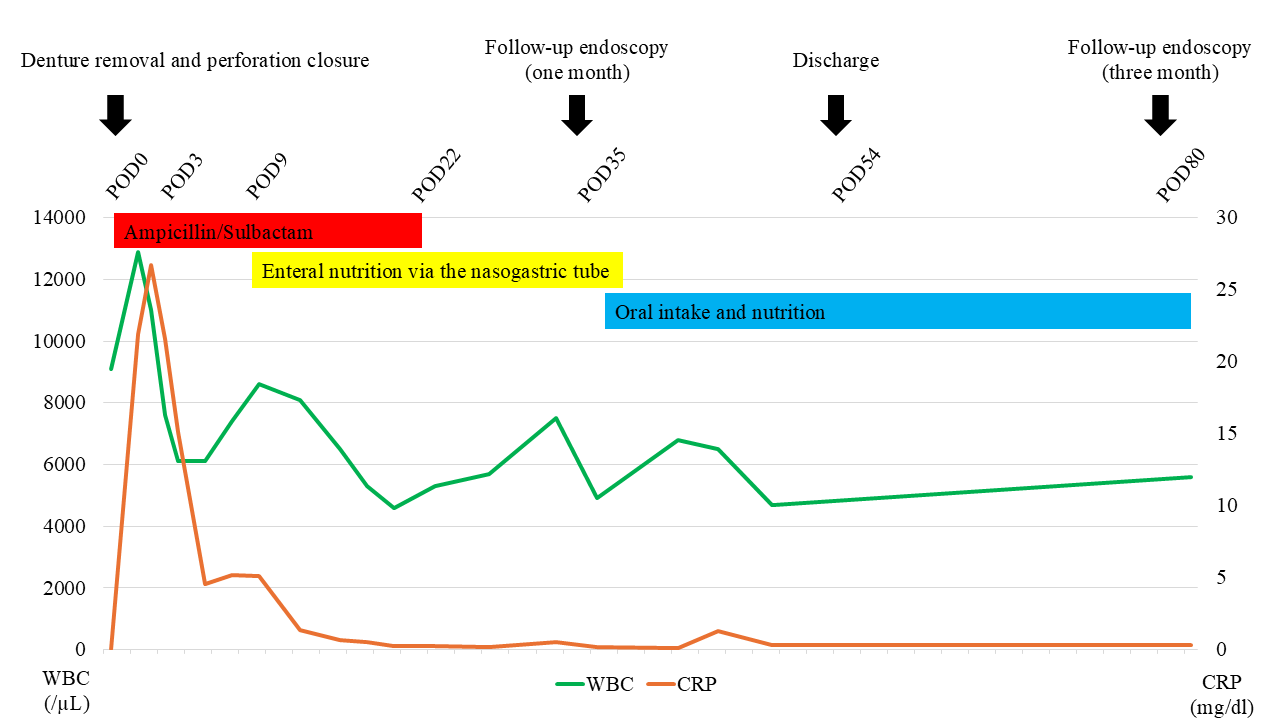

Supplement: Supplementary file 2 — Figure S2: Clinical course summarizing the timeline of symptoms, treatment procedures, and laboratory data (C‐reactive protein [CRP] level and white blood cell [WBC] count). [file DEO2-6-e70270-s001.tif]
